# Aggressive T1 vertebral haemangioma initially thought to be plasmacytoma: lessons from CT–MRI correlation

**DOI:** 10.1093/omcr/omag034

**Published:** 2026-04-14

**Authors:** Zack Bin Azlin, Selnan J Wuyep, Andreas K Demetriades

**Affiliations:** Edinburgh Spinal Surgery Outcome Studies Group, Department of Neurosurgery, Royal Infirmary of Edinburgh, Little France Crescent, Edinburgh EH16 4SA, UK; Royal Infirmary of Edinburgh, Little France Crescent, Edinburgh EH16 4SA, UK; Edinburgh Spinal Surgery Outcome Studies Group, Department of Neurosurgery, Royal Infirmary of Edinburgh, Little France Crescent, Edinburgh EH16 4SA, UK; Royal Infirmary of Edinburgh, Little France Crescent, Edinburgh EH16 4SA, UK; Edinburgh Spinal Surgery Outcome Studies Group, Department of Neurosurgery, Royal Infirmary of Edinburgh, Little France Crescent, Edinburgh EH16 4SA, UK

**Keywords:** vertebral haemonagioma, CT-MRI correlation, polka-dot appearance, radiological cord compression, plasmacytoma, clinicoradiological surveillance

## Abstract

The reduced specificity of certain MRI findings for aggressive vertebral haemangiomas (VH) may result in uncertainty surrounding the diagnosis. Utilising concurrent CT scans in such cases may provide additional evidence to help improve diagnostic accuracy. We report a case of a 68-year-old woman presenting with an aggressive vertebral haemangioma variant where the importance of CT-MRI correlation is demonstrated.

## Introduction

Vertebral haemangiomas (VH) are the most common benign spinal tumours and are identified incidentally in an estimated 10%–12% of adults [1]. Typical fat-rich phenotypes have characteristic appearances on CT and MRI [1–3]. However, atypical or aggressive variants may present as fat-poor and with epidural extensions, an appearance on MRI which can overlap with a possible plasmacytoma or metastasis [1, 3, 4]. In such cases, CT imaging showing coarse vertical trabeculae with axial ‘polka-dot’ foci strongly favours the diagnosis of VH [2]. We report a case of cervicothoracic VH initially diagnosed as probable plasmacytoma based on suggestive MRI features, later reclassified following CT imaging. This case highlights how intentional CT-MRI correlation can avoid unwarranted escalation into oncologic pathways and justify a surveillance approach [1–3].

## Case report

**Figure 1:**
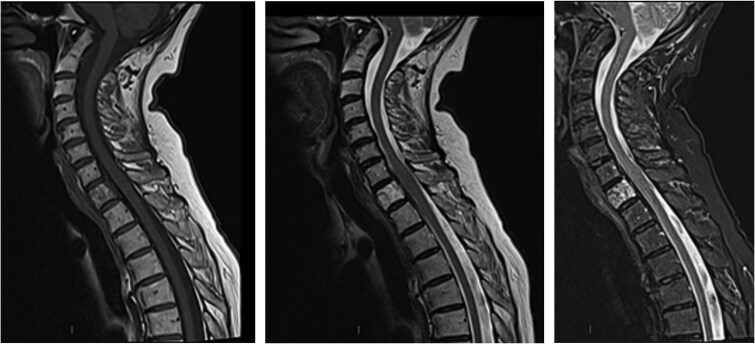
T1W, T2W, STIR sagittal MRI imaging demonstrating a vertebral haemangioma at the level of the first thoracic vertebra. No loss of vertebral body height or alignment.

**Figure 2:**
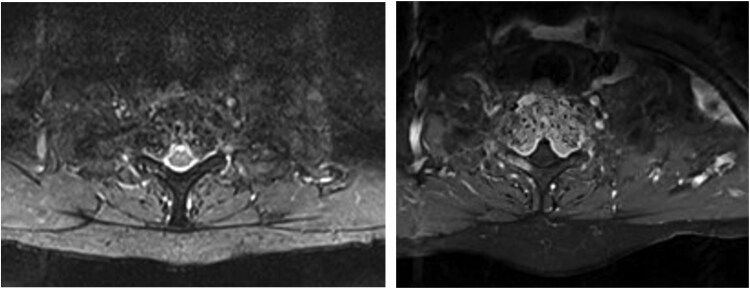
T2W and T1W FS axial MRI images showing changes extending into bilateral pedicles and bulging cortical margins. The vertebral canal is narrowed by the extradural haemangioma component, with distortion but not compression of the spinal cord. Cord signal is normal. There is bilateral compression of the T1 nerve roots.

A 68-year-old woman presented with several months of neck and interscapular pain, which intermittently radiated towards her upper limbs. No constitutional symptoms were reported. Physiotherapy noted mixed cervical radicular symptoms with morning hand paraesthesiae suggestive of carpal tunnel syndrome, and advised imaging if symptoms persisted. Symptoms were managed with simple analgesia, without neurological progression. On further presentation to the GP, she was subsequently referred for spinal imaging. An initial MRI of the cervicothoracic junction described a lesion centred at the T1 vertebral body with bony expansion, severe bilateral T1/2 foraminal stenosis, and spinal canal narrowing. The patient’s CSF around the cord was preserved with no intramedullary cord signal change identified (see Figs 1 and 2). The initial radiological impression was probable solitary plasmacytoma, and urgent haematology and neurosurgery review was suggested.

**Figure 3:**
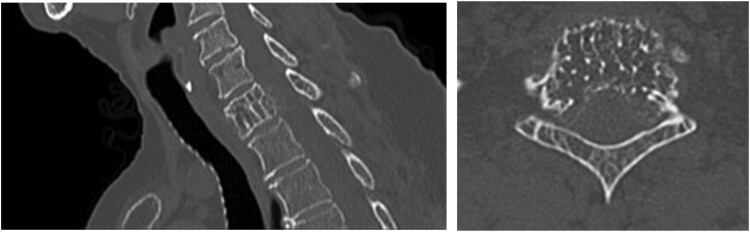
CT scan in sagittal and axial views through the first thoracic vertebra. The components which extend into the epidural space, bilateral T1 neural exit foramen and inferomedial aspect of the left C8 neural foramen contain thin calcific/ossific trabeculations. Vertebral height is maintained, and alignment is normal.

A subsequent CT demonstrated a predominantly low-attenuation lesion within the T1 vertebral body containing thickened craniocaudal (vertical) trabeculae with an enhancing epidural component extending posteriorly into the canal and subarticular recesses, narrowing the canal and foramina (see Fig. 3). These appearances are typical of a VH (aggressive phenotype) [1, 2]. An addendum to the MRI revised the working diagnosis to haemangioma, once the CT scan features were considered. A planned haematology clinic was subsequently stood down as ‘not haematologically relevant’. Neurosurgical clinic review found no myelopathic signs, and surveillance imaging was planned. A contrast-enhanced MRI of the craniocervical junction to T5, six months later, confirmed a T1 VH with extension into both pedicles and bulging cortical margins. There was a slight interval increase in intraspinal epidural extension with greater indentation of the cord and loss of posterior CSF at T1; vertebral height and alignment were preserved, and there remained no intramedullary cord signal change. A myeloma screen, including serum and urine electrophoresis, and serum free light chains, was also arranged after the initial MRI and yielded no abnormalities that would require any haematologic treatment. No biopsy was performed due to concerns about the risk of potential bleeding.

The patient was referred to the interventional neuroradiology multi-disciplinary meeting; it was decided that there was no evidence to support stand-alone embolisation in this context and deemed that the lesion’s position and extra-osseous extension rendered it unsuitable for endovascular treatment. Therefore, the patient has been managed conservatively, with safety-netting advice and closer-interval MRI access if symptoms or imaging changed.

## Discussion

This case demonstrates how CT–MRI correlation resolved an MRI-driven malignant differential and guided proportionate management [1–3]. Fat poor or ‘aggressive’ VH may demonstrate enhancement, marrow oedema, and epidural or extraosseous extension on MRI, features that can overlap with those of plasmacytoma or metastatic disease and often prompt oncologic evaluation [1, 3, 4]. In such settings, CT features (corduroy trabeculae; axial ‘polka-dot’ foci) strongly support VH and should be explicitly correlated in reports [1, 2]. In our patient, this correlation overturned an initial working diagnosis of ‘probable solitary plasmacytoma,’ halted an unnecessary haematology work-up, and refocused care on neurosurgical follow-up.

Similar diagnostic challenges have been described in wider literature, where atypical vertebral haemangiomas may mimic malignant lesions. One report described a plasmacytoma misinterpreted as atypical vertebral haemangioma on MRI; CT demonstrated lytic change and corrected the diagnosis [5]. CT correlation is valuable; trabecular changes may be subtle on MRI. Multidisciplinary input can help in complex cases requiring angiography or biopsy. Reported management ranges from surveillance to embolisation and/or surgery ± vertebroplasty [6].

In-phase and out-of-phase MRI has a limited but specific role in evaluating ‘fat-poor’ atypical hemangiomas by assessing intralesional fat. These lesions demonstrate reduced or absent signal, reflecting a lack of macroscopic fat and assisting differentiation from metastases, which typically lack fat but may show differing enhancement and diffusion characteristics. Quantitative signal-loss analysis can improve diagnostic accuracy, with up to 84.88% reported differentiation accuracy [7]. However, T1-weighted imaging with and without fat suppression remains the most sensitive and reliable method for distinguishing hemangiomas from metastases.

Another teaching point concerns risk stratification. A minority of VH behave ‘aggressively,’ with epidural extension, cortical expansion, canal and foraminal stenosis, and occasionally vertebral collapse [1]. Even so, cord indentation alone is not synonymous with myelopathy. When the intramedullary cord signal is preserved and the neurological examination remains stable, surveillance is a fair strategy, reserving intervention for progressive pain, new deficit, structural instability/collapse, or emergence of cord signal change [1]. This is consistent with our patient’s course; slight interval increase in the epidural component and greater indentation yet preserved cord signal and a stable examination over > 12 months.

Finally, therapy is anatomy and symptom dependent. Options for symptomatic/progressive VH include decompression ± pre-operative embolisation and vertebroplasty/kyphoplasty, selected by neural compression, stability, vascularity, and access corridors [1, 6–8]. Evidence for stand-alone embolisation in asymptomatic or minimally symptomatic VH is limited, and cervicothoracic anatomy may preclude a safe endovascular approach [8–10].

This case highlights a clear diagnostic pathway at the cervicothoracic junction, an area prone to artefact. Deliberate CT–MRI correlation can prevent mislabeling, reduce low-yield referrals, and support watchful waiting when there are no objective signs of myelopathy.

CT–MRI correlation reclassified a presumptively malignant T1 lesion as a vertebral haemangioma [1–4]. With cord indentation but no intramedullary signal change and a stable clinical examination, surveillance has been appropriate while monitoring for clinical or radiological progression [1].
